# Palladium/Carbon Nanofibers by Combining Atomic Layer Deposition and Electrospinning for Organic Pollutant Degradation

**DOI:** 10.3390/ma13081947

**Published:** 2020-04-21

**Authors:** Melissa Najem, Amr A. Nada, Matthieu Weber, Syreina Sayegh, Antonio Razzouk, Chrystelle Salameh, Cynthia Eid, Mikhael Bechelany

**Affiliations:** 1 Institut Européen des Membranes, IEM–UMR 5635, ENSCM, CNRS, University Montpellier, 34730 Montpellier, France; melissa-najem@hotmail.com (M.N.); chem_amr@yahoo.com (A.A.N.); matthieu.weber@umontpellier.fr (M.W.); syreina.sayegh@gmail.com (S.S.); Chrystelle.Salameh@enscm.fr (C.S.); 2 Department of Analysis and Evaluation, Egyptian Petroleum Research Institute, Cairo, Nasr City P.B. 11727, Egypt; 3 Laboratory of Chemical Analyses, Faculty of Sciences 2, Lebanese University, Fanar B.P. 90656, Lebanon; carlorazzouk@hotmail.com; 4 EC2M, Faculty of Science 2, Fanar Campus, Lebanese University, Fanar B.P. 90656, Lebanon; cynthia.eid@ul.edu.lb

**Keywords:** palladium nanoparticles, carbon nanofibers, nanocatalysts, electrospinning, atomic layer deposition, heterogeneous catalysis, methyl orange degradation, catalysts recovery

## Abstract

As organic dyes are a major source of pollution, it is important to develop novel and efficient heterogeneous catalysts with high activity for their degradation. In this work, two innovative techniques, atomic layer deposition and electrospinning, were used to prepare palladium nanoparticles (Pd NPs) supported on carbon nanofibers (CNFs). The sample morphology was investigated using scanning and transmission electron microscopy. This showed the presence of nanofibers of several micrometers in length and with a mean diameter of 200 nm. Moreover, the size of the highly dispersed Pd NPs was about 7 nm. X-ray photoelectron spectroscopy visually validated the inclusion of metallic Pd. The prepared nano-catalysts were then used to reduce methyl orange (MO) in the presence of sodium borohydride (NaBH_4_). The Freundlich isotherm model was the most suitable model to explain the adsorption equilibrium for MO onto the Pd/CNF catalysts. Using 5 mL MO dye-solution (0.0305 mM) and 1 mL NaBH_4_ (0.026 mM), a 98.9% of catalytic activity was achieved in 240 min by 0.01 g of the prepared nano-catalysts Pd/C (0.016 M). Finally, no loss of catalytic activity was observed when such catalysts were used again. These results represent a promising avenue for the degradation of organic pollutants and for heterogeneous catalysis.

## 1. Introduction

Dyes are extensively used particularly by textile industries for color enhancement; however, they represent a major source of pollution. Dyes are responsible not only for major ecosystem contaminations, but they might also have serious consequences on human health because they are currently used in textiles, food, plastics, printing and cosmetics [1]. More than 7 × 10^5^ tons of synthetic dyes are produced every year, and the majority of non-biodegradable textile dye effluents are discharged in natural water streams and water systems [2]. This represents up to 1000 tons of waste per year. In addition, dye waste management is particularly difficult due to their high resistance to biological and physical treatments, and sometimes the degradation products are even more toxic [3]. Consequently, cheap and effective “green” systems to remove dyes from water effluents are required.

Recently, many groups have investigated the application of heterogeneous catalysis (an effective and low-cost method) for the degradation of organic pollutants, and have tried to fabricate new heterogeneous catalysts for environmental applications, including dye degradation.

Catalysts can be reused many times because they are typically very stable [1,4]. Previous studies described the successful application of noble-metal nanoparticles (NPs), such as palladium, platinum, rhodium and ruthenium, as catalysts in many different fields, particularly chemical synthesis, oil refinement, hydrogen storage, exhaust gas treatment, and sewage treatment [5,6,7,8,9,10,11]. The high efficiency of such NPs is explained by their high values of specific surface area, highly ordered structure, high density of coordination sites, elevated oxidation activity, and outstanding mechanical and thermal stability [1]. The catalytic features of metallic NPs are influenced by their composition, shape and size [12]. Palladium (Pd) nanomaterials are important for many chemical reactions [1]. For example, Pd can be used as catalyst to facilitate hydrogen absorption and detection due to its high affinity for absorbing hydrogen [5,6,7,8,10,13]. Moreover, Pd nanoparticles are more effective in removing dyes from aqueous solutions by heterogenous catalysis than many typically used methods, such as chemical precipitation, filtration, adsorption and biological treatments. The industrial applications of these nanoparticles are, however, still hindered due to their high cost and limited resources. To overcome this issue, immobilizing noble-metal nanoparticles with small dimensions on high surface-to-volume ratio solid supports, such as carbon materials is regarded as an effective strategy [14].

The choice of supporting material on which NPs are deposited is of fundamental importance, because it will play a major role in the stabilization of the active catalytic phase. Moreover, the transport of reactants to the active sites can be controlled by modulating the supporting material porosity. Therefore, microporous carbon is the first choice of support material for noble-metal NPs with catalytic activity [4,15].

Atomic layer deposition (ALD) is an innovative strategy that allows growing thin films and NPs while controlling their nanoscale dimensions [16,17,18]. In ALD, sequential and self-limiting chemical reactions, which form a closed cycle, allow the synthesis of different inorganic nanomaterials, for instance oxides [19,20] nitrides [21,22] and metals [23,24]. In a typical ALD cycle, a pulse of precursor is followed by purging and then by a pulse of co-reactant (i.e., gaseous molecules) [25,26]. The control of thickness at the atomic level and the very good uniformity and conformality across the substrate surface are the main advantages of ALD. They also explain why ALD is more and more used for nanofiber coating [25,27,28,29,30], and for the deposition of NPs and thin films, especially for microelectronics [31,32] membranes [16,33,34,35] and catalysis [11,36,37,38]. Carbon nanomaterials are frequently chosen as solid supports for catalysts because they have a large surface area, very good heat conductivity and exceptional mechanical properties [4,39].

Electrospinning is an attractive route for their preparation. This is a well known, low-cost method in which electrostatic forces are used for nanofiber production [40,41,42,43,44,45,46,47,48]. Furthermore, electrospinning allows modulating the surface-to-volume ratio and porosity and also controlling the size and morphology in order to produce nanofibers that display the best features for the desired application [49]. Thus, electrospun carbon nanofibers present numerous advantages due to their outstanding properties such as high electrical conductivity, chemical inertness, and physical and mechanical stabilities [50,51]. The nanofibers could be also be modified by adding functional groups or combined with conducting nanomaterials like metals to generate hydrophilic surfaces [52]. Moreover, compared to other preparation techniques, it is possible to produce the carbon nanofibers by vapor grown carbon with a similar size scale, but the obtained filaments are discontinuous and not well oriented in comparison to the continuous electrospun carbon nanofibers [43,50]. We note as well that polyacrylonitrile (PAN) nanofibers obtained by electrospinning could be carbonized 1000 times faster than conventional fibers; thus, that will be a promising lower-cost novel material [43]. In agreement, several studies have described the application of electrospun nanofibers for air filtration [53] and as templates for producing tubular nanostructures (e.g., metal nanotubes [54], metal oxide nanotubes [55], and polymer nanotubes [56]). Electrospun fibers represent a precious material also for tissue engineering [57], drug delivery systems [58], protective clothing [59], filtration media [60], and charge storage devices such as supercapacitors and novel batteries [61,62]. An electrospinning device can be easily built. It consists of a high-voltage electrical source of positive and negative polarity, a syringe with a metal needle, a syringe pump, and a conductive collector. Typically, an electrical potential is applied between metal needle and collector. When the electric field overcomes the droplet surface tension, a charge jet of polymers appears as a Taylor cone and its trajectory is controlled by the applied electric field. The jet extends through a spiral loop and eventually, nanofibers are collected on the conductive target covered by a thin film of aluminum. [43].

Here, Pd NPs deposited on carbon nanofibers (CNFs) were synthetized by combining electrospinning and ALD. These materials were employed to reduce methyl orange (MO) in the presence of sodium borohydride (NaBH_4_). The MO dye was chosen for these tests because azoic dyes represent 50% of all dyes used by industries [3]. The catalytic activity of the prepared nanomaterials was assessed by monitoring MO degradation in normal conditions. Our results might promote more research to develop more effective and cheap adsorption methods for dye removal and wastewater treatment, as in [63,64,65,66].

## 2. Materials and Methods

### 2.1. Chemicals

Palladium (II) hexafluoroacetylacetonate {(Pd(hfac)_2_), CAS number: 64916-48-9, 95%} and formalin {neutral buffered, 10%, CAS number: 50-00-0} were used as precursor and co-reactant for ALD of Pd. N,N-dimethylformamide {DMF, CAS number 68-12-2, 99.8%) and polyacrylonitrile {PAN, CAS number: 25014-41-9, MW = 500,000} were employed to prepare PAN nanofibers. MO {CAS number: 547-58-0, 85%} was the pollutant used to assess the photocatalytic activity of our samples. Sodium borohydride {NaBH_4_, CAS number:16940-66-2, 99.99%} was used as a base and to control the solution pH. All chemicals were from Sigma-Aldrich (Saint-Quentin Fallavier, France) and were used without any additional purification.

### 2.2. Synthesis of Palladium/Carbon Nanofibers

#### 2.2.1. CNFs

To obtain carbon fibrous nanomaterials, PAN nanofibers were prepared by electrospinning. PAN was mixed with DMF (2 g PAN/18 mL DMF) by stirring at 60 °C for 24 h and then the solution was electrospun at 30 ± 2 °C and 25 kV of applied voltage, with a solution flow rate of 3 mL/h and a tip-collector distance of 15 cm. PAN fibers were collected on a spinning cylinder (10 cm in diameter) with a rotating speed of 230 rpm covered with aluminum foil. The process lasted 7 h. Then, PAN nanofibers underwent stabilization in an air furnace for 2 h (at 250 °C, heating rate of 1 °C/min), followed by carbonization (1000 °C for 1 h) in a controlled atmosphere furnace (high purity nitrogen; heating rate of 2 °C/min). The color of PAN fibers changed from white (just after electrospinning) to brown (after stabilization) to black (i.e., electrospun CNFs).

#### 2.2.2. ALD of Pd on CNFs

For Pd NP deposition on electrospun CNFs, a low-pressure hot-wall (home-built) ALD system was used.

The first step was heating the precursor (Pd(hfac)_2_) and co-reactant (formalin) lines to 70 °C and 100 °C to avoid condensation, and the precursor bubbler to 69 °C to improve the arrival of Pd vapor into the deposition chamber because Pd has a low vapor pressure. The temperature of the deposition chamber was 220 °C. For Pd deposition on CNFs, the ALD cycle was as follows: Pd(hfac)_2_ pulse for 5 s, exposure for 15 s, purge for 20 s, formalin pulse for 5 s, exposure for 15 s, purge for 20 s, and purge with Argon for 60 s (200 cycles, if not otherwise stated).

### 2.3. Characterization of Samples

The morphology of the synthesized CNFs and Pd on CNFs materials was examined by scanning electron microscopy (SEM, ZEISS EVO HD-15, ZEISS, Marly le Roi, France). A JEOL 2200FS (JEOL, Akishima, Tokyo, Japan) field emission transmission electron microscope (TEM) operated at 200 kV was used for high-resolution morphological analyses. Elemental mapping was obtained using energy-dispersive X-ray (EDX) and an EDAX (Energy Dispersive Analysis of X-rays) attachment (Oxford Instruments, High Wycombe, UK).

The surface state was characterized by X-ray photoelectron spectroscopy (XPS) with an ESCALAB 250 spectrometer (Thermo Electron, monochromatic radiation source Al Kα = 1486.6 eV, Thermo Fisher Scientific, Waltham, MA, US). Survey spectrum recording was performed at 1 eV steps (transition energy: 150 eV) and high-resolution spectra were acquired at 0.1 eV steps (transition energy: 20 eV).

A dispersive Raman microscope (Model Sentera, Bruker, Germany) with a laser wavelength of 532 nm and a 20/50 mW (Nd:YAG) laser was used for molecule identification.

The crystalline phase of the prepared CNFs and Pd on CNF samples was analyzed by X-ray diffraction (XRD) with a PANAlytical Xpert-PRO diffractometer (Malvern Panalytical, Malvern, UK) equipped with an X’celerator detector (Malvern Panalytical, Malvern, UK) and using Ni-filtered Cu-radiation (λ = 1.54 Å).

The MO dye concentrations were calculated on the basis of the UV–Vis (UltraViolet-Visible) absorbance (spectra acquired with a Jasco V-570 UV–VIS-NIR spectrophotometer (Jasco Inc., Easton, PA, USA) at λ = 464 nm) and the external calibration method.

### 2.4. Catalytic Activity

MO was chosen as typical organic pollutant to reduce. For this purpose, a stock solution of 10 ppm MO (0.0305 mM) and a solution of 1 ppm NaBH_4_ (0.026 mM) were prepared. The sample catalytic activity of 0.01 g catalyst was tested in 10 mL flasks that contained 5 mL of aqueous dye solution with increasing MO concentrations of 10, 15, 20, 25, 30 ppm (0.0305, 0.04582, 0.061, 0.0763 and 0.09165 mM) and 1 mL of NaBH_4_ solution and 0.01 g of the catalyst; thus, the catalyst-dye ratio was maintained at 2 g/L (0.016 M). Flasks were placed on a magnetic stirrer (400 rpm) at room temperature. MO degradation in the presence of CNFs and Pd/CNFs was monitored by collecting samples of the dye solution at different time points (0, 30, 60, 90, 120, 150 and 180 min). The UV–Vis absorbance spectra of the MO solution displayed a main absorption band at 464 nm. Isothermal and kinetic analyses were carried out to understand the degradation process.

#### 2.4.1. Adsorption Isotherms

The isotherms of MO dye adsorption on Pd/CNFs and CNFs were generated using the Langmuir and Freundlich theoretical models. The Langmuir model hypothesizes that a monolayer of adsorbate molecules surrounds the adsorbent homogeneous surface. In this model, interactions between adsorbate molecules are considered insignificant [63,64]. On the other hand, the Freundlich model implies a multilayered adsorbate and an adsorbent with a non-homogeneous surface [63,64].

The Langmuir isotherm linear form was plotted with Equation (1):

(1) $$\frac{C_{e}}{q_{e}}=\frac{C_{e}}{q_{\max}}+\frac{1}{q_{\max\;}{\;K}_{L}}$$

where q_max_ (mg/g) and k_L_ (L/mg) represent the Langmuir isotherm constants that describe the maximum adsorbed quantity and the free energy function of adsorption, respectively.

The linear form of the Freundlich model was plotted with Equation (2):

(2) $$\ln\;qe_{\;}=\ln\;K_{F}+\frac{1}{n}\;\ln\;C_{e}$$

where the Freundlich isotherm coefficients K_F_ ((mg/g)(L/mg)^1/n^) and n illustrate the adsorption capacity and adsorption intensity.

#### 2.4.2. Adsorption Kinetics

To determine MO adsorption kinetics on Pd/CNFs and CNFs and to describe the link between adsorbed quantity and sorption time, different kinetic models were used [63].

The MO adsorption kinetic data were analyzed with the linear equations of the pseudo-first-order (Equation (3)) and pseudo-second-order models (Equation (4)).

(3) $$\log\;(q_{e}-q_{t})=\log\;q_{e}-\frac{K_{1}}{2.303}\;t$$

where K_1_ (1/min) is the kinetic rate constant for the pseudo-first-order adsorption model.

(4) $$\frac{t}{q_{t}}=\;\frac{1}{K_{2}q_{e\;}^{2}}+\frac{1}{q_{e}}\;t$$

where K_2_ (g/(mg·min)) denotes the rate constant for the pseudo-second-order adsorption model.

## 3. Results and Discussion

### 3.1. Characterization of Palladium/Carbon Nanofibers

#### 3.1.1. Morphological Properties of CNFs and Pd/CNFs

**Figure 1:**
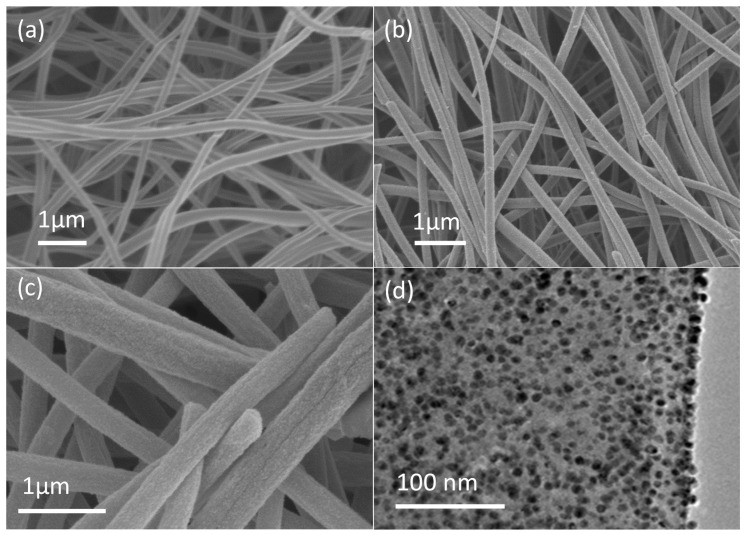
(**a**–**c**) SEM images showing CNFs and Pd/CNFs, (**d**) TEM image of Pd/CNFs.

Figure 1a shows SEM (Scanning Electron Microscopy) photographs of electrospun CNFs after heat treatment of PAN NFs under air and under nitrogen. This figure highlights the CNF uniformity (mean CNF diameter = 207 ± 40 nm), the highly interrelated networks of continuous and randomly oriented NFs, and the smooth external surface. The absence of visible defects indicates the good quality of the samples. For CNF fabrication, a PAN polymeric solution was electrospun, followed by heat treatment at high temperature. Pd NPs were then deposited by ALD on the electrospun CNFs.

As SEM resolution is limited, TEM (Transmission Electron Microscopy) was then used to thoroughly study the morphology and structure of Pd NPs deposited on CNFs (200 ALD cycles). This analysis confirmed the presence of Pd NPs (black dots Figure 1d) and their homogeneously dispersion (>70% coverage). The mean diameter of Pd NPs was 7.305 ± 1.53 nm (Figure 1d). Pd particles were homogeneously dispersed on the carbon substrate (Figure 1c,d at higher magnification), thus maximizing the catalytic activity of the CNF supports.

**Figure 2:**
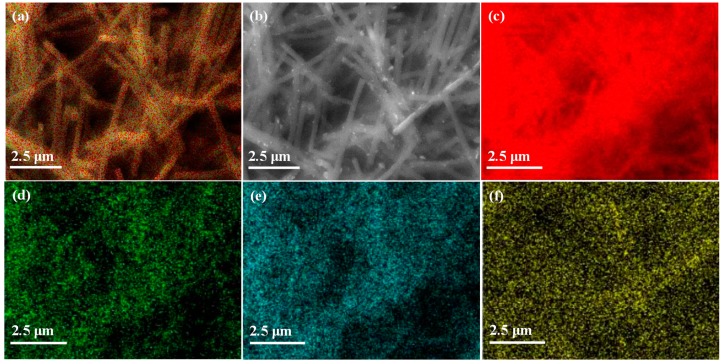
Elemental mapping and distribution of all elements. (**a**), Electron microscopy image (**b**), and images of each element: (**c**) C, (**d**) N, (**e**) O, and (**f**) Pd in Pd/CNFs.

Figure 2 shows the results of the EDX (energy-dispersive X-ray) analysis of Pd/CNFs and of elemental mapping.

The quantitative EDX analysis gave the following molar ratios: Pd (1.0% ± 0.4%), C (80.8% ± 1.5%), N (14.7% ± 0.3%), O (3.5% ± 1.1%), and the following weight ratios: 72.8 ± 3.6 wt% of C, 15.1 ± 0.7 wt% of N, 3.8 ± 1.08 wt% of O, 8.3 ± 3.2 wt% of Pd. Elemental mapping (Figure 2) confirmed that the material was mainly constituted of carbon and that Pd was deposited as NPs by ALD, validating the TEM results.

#### 3.1.2. Surface Properties of Pd/CNFs

**Figure 3:**
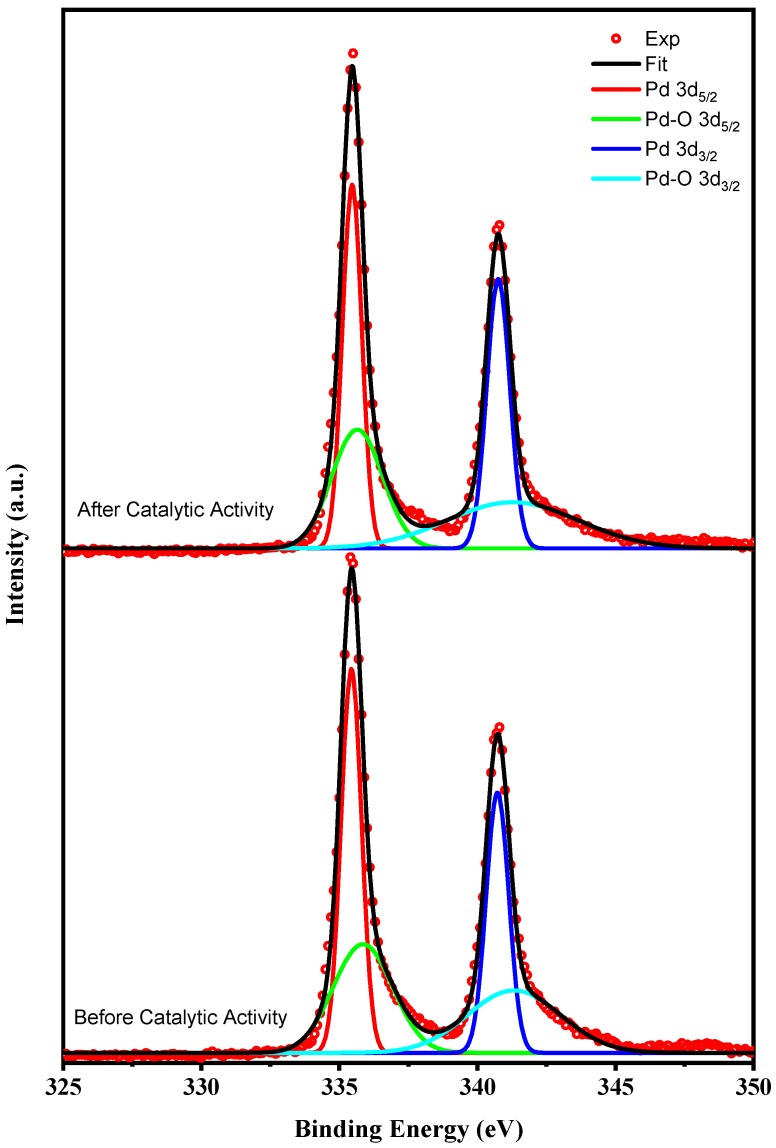
High-resolution XPS spectra of Pd 3d core level.

XPS (X-ray photoemission spectroscopy) was used to determine the sample surface state. The binding energy (corrected on the basis of C 1s binding energy at 284.4 eV) was quantified from the corresponding XPS peak area after correction using a relevant sensitivity factor. Figure 3 presents the XPS spectra of Pd 3d for Pd/CNFs (200c). The reason why Pd is stabilized on CNF is that the metallic atoms are not physically adsorbed, but chemically bonded to the substrate. In addition, the porosity of the CNF substrate enables for a higher aspect-to-surface ratio and thus, a higher loading of the Pd nanoparticles.

Figure 3 indicates the presence of two chemical states of Pd. The Pd metal doublets were at 335.5 eV (Pd 3d_5/2_) and 340.7 eV (Pd 3d_3/2_). After heating, Pd NPs were easily oxidized to palladium oxide. The components with the lower and higher binding energy could represent metallic Pd and PdO after partial oxidation of the Pd NP surface, respectively. The PdO/Pd ratio was 0.83 before and after catalytic activity. Clearly, Pd NPs promote CNF catalytic activity. [67]

#### 3.1.3. CNF and Pd/CNF Structural Properties

**Figure 4:**
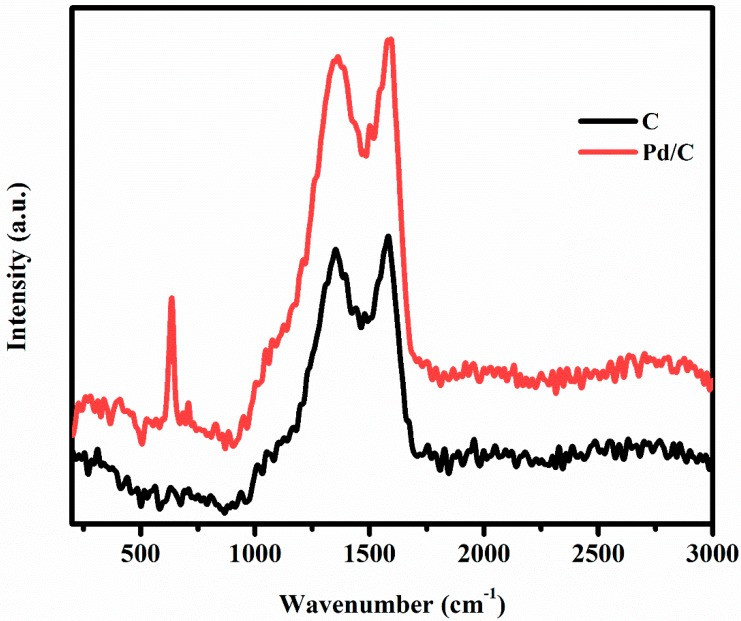
Raman spectra of CNFs and Pd/CNFs.

To identify the individual components and to analyze the effect of Pd NP deposition on CNFs, Raman spectroscopy was carried out. Figure 4 shows the spectra of CNFs with and without Pd NPs (red and black curves, respectively). It can be clearly noticed that carbon remained intact before and after Pd deposition. On the other hand, a new peak was observed in the Pd/CNFs sample. On the basis of the results of previous studies [68,69], this peak, which displays a shift of about 649 cm^−1^, was linked to the presence of PdO in CNFs.

The crystalline phases of CNF and Pd/CNF diffraction patterns of the activated carbons were studied by XRD.

**Figure 5:**
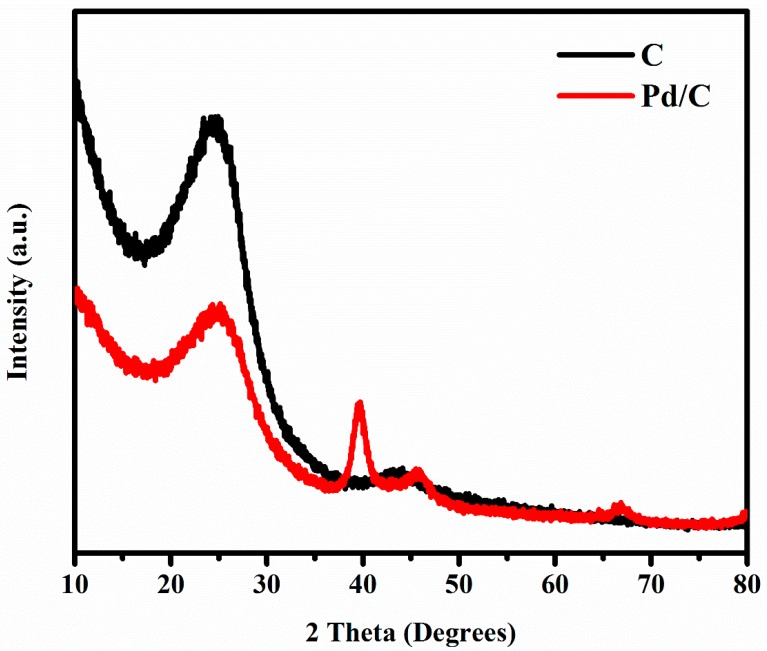
XRD results of CNFs and Pd/CNFs.

The CNF profile (black curve in Figure 5) shows a diffraction peak around the 2θ of 25°–26° attributed to graphite crystallite (002) crystallographic plane [70]. The Pd/CNF profile (red curve in Figure 5) confirms the Pd phase deposition on CNFs by ALD. The XRD pattern can be explained by the typical face-centered cubic (fcc) lattice structure of Pd, according to the standard values reported for Pd. The strong diffraction peak at the Bragg angle of 2θ = 40° refers to the diffraction of the (111) plane [71,72]. The diffraction peaks close to 26° and 47° could be the diffraction of the (002) and (100) planes of carbon [71].

Based on Scherrer equation (Equation (S1) in supplementary materials), according to which the NP size in a solid is related to the broadening of a peak in the diffraction profile, an intuitional analysis was done for Pd NPs to determine the crystallite size from the XRD pattern (Figure 5) [39].

After studying the 40° diffraction peak of the Pd/CNF sample (red curve in Figure 5), the mean size was estimated at 7.4 ± 0.4 nm. This result is in agreement with the mean diameter of 7.305 ± 1.53 nm for Pd NPs calculated from Figure 1d.

### 3.2. Catalytic Activity Results

**Figure 6:**
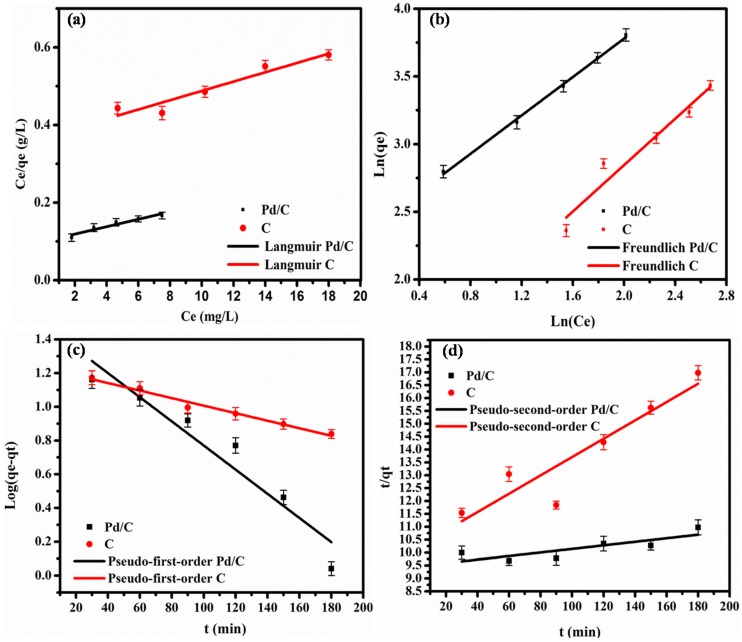
Linear Langmuir model (**a**) and linear Freundlich model (**b**) for MO dye adsorption onto Pd/CNF and CNF samples after 3 h. The curves show the linear fitting of the experimental data. Linear fitting of the kinetic results using the pseudo-first-order (**c**) and pseudo-second-order (**d**) models during 3 h.

The crucial point was to understand the adsorption process to evaluate the catalytic activities of CNF and Pd/CNF as catalysts for MO degradation in normal conditions. For this reason, isothermal and kinetic studies were carried out based on the UV–Vis (UltraViolet-Visible) spectrophotometer data, while the catalytic activity was tested three times, and at each run, adequate degradation data were collected and studied. The error bar indicates its range of variation in the isothermal and kinetic graphs; this is well-illustrated in the figure bellow (Figure 6) for both Pd/CNF surface and the CNF surface separately, during 3 h.

#### 3.2.1. Isothermal Study

The experimental data were fitted using isotherm models and linear regression analysis (Figure 6a,b). The table below (Table 1) summarizes the constants and the adsorption parameters (calculated using Equations (1) and (2), described in Methods), and the coefficients R^2^ of the fitting.

The Langmuir model (Figure 6a) gave lower R^2^ values for both Pd/CNF and CNF catalysts (0.932 and 0.922, respectively) than the Freundlich model (Figure 6b), (0.998 and 0.950 for Pd/CNF and CNF). This indicates that the equilibrium adsorption process of MO onto Pd/CNF and CNF is better described by the Freundlich model (Table 1). In conclusion, it is likely that MO is adsorbed on the adsorbent non-homogeneous surface via a multilayer adsorption process.

#### 3.2.2. Kinetic Study

The pseudo-first-order and pseudo-second-order kinetic models were used to determine the effect of the contact time on MO adsorption onto Pd/CNF and CNF at room temperature, and the adsorption mechanism, respectively (Figure 6c,d). The equations of these two models (Equations (3) and (4)) allowed us to determine the kinetic constants and the coefficients R^2^ (Table 2).

Figure 6c and Table 2 clearly indicated that the linear form of the pseudo-first-order model better describes the experimental kinetic data (R^2^: 0.92 and 0.98 for Pd/CNF and CNF) than that of the pseudo-second-order model (R^2^: 0.67 and 0.86 for Pd/CNF and CNF as catalysts).

These results show that MO is adsorbed on a heterogeneous surface via a multilayer adsorption process because the equilibrium adsorption process could be better fitted with the Freundlich model, and the calculated K_f_ was 10.60 (mg/g)(L/mg)^1/n^ for Pd/CNF as catalyst. The adsorption kinetic results were well described by the pseudo-first-order model.

**Figure 7:**
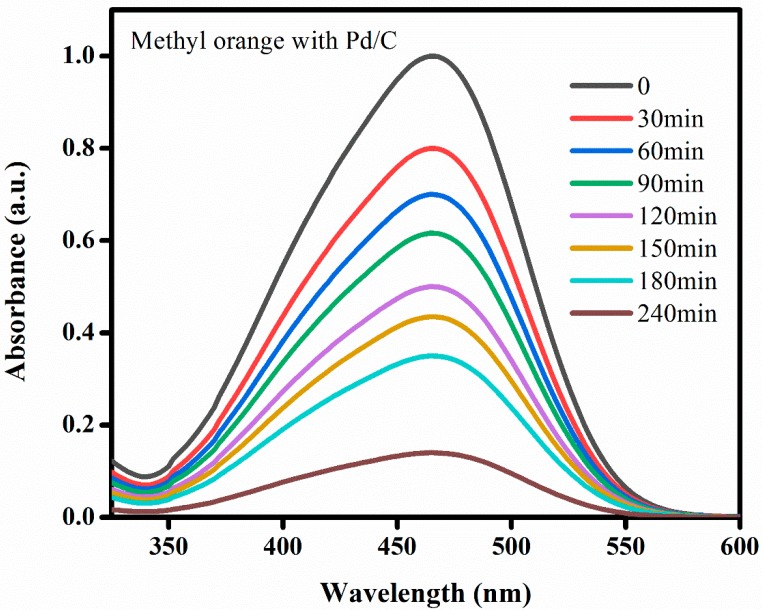
UV–Vis spectra of MO degradation with the Pd/C as catalyst.

As illustrated in Figure 7 below, after 4 h of catalyst-dye contact, 98.9% of MO was degraded. Indeed, many research studies have been dedicated to investigating MO degradation [1,3,65,73,74,75,76,77,78]. However, only one paper reported the deposition of Pd on one dimensional carbon materials (Carbon nanotubes (CNTs)) and their as catalyst for the MO degradation [1]. Where 2 g/L was also the adsorbent-dye dose (0.016 M as catalyst concentration), the final degradation process efficiency of Pd/CNTs as catalyst, comparing to our results, may be surprising. Yet, according to that previous study, there were main differences, as clearly detailed in Table 3.

Thus, as seen in Table 3, by comparing our results with those in the previous cited reference on MO-degradation capacities of Pd/C, it is clear that we came by a high degradation percentage (98.9%) with a small amount of Pd NPs deposited on the same support (carbon). This is definitely a spotlight on the competitive efficiency of these ALD-deposited NPs with smaller-amount and smaller-mean diameter. Moreover, as mentioned in the literature, Pd NPs could be used for the degradation of several dyes such methylene blue, Congo red, 4-Nitrophenol, Methyl orange, Sunset yellow and Tartrazine [14,80,81,82].

**Figure 8:**
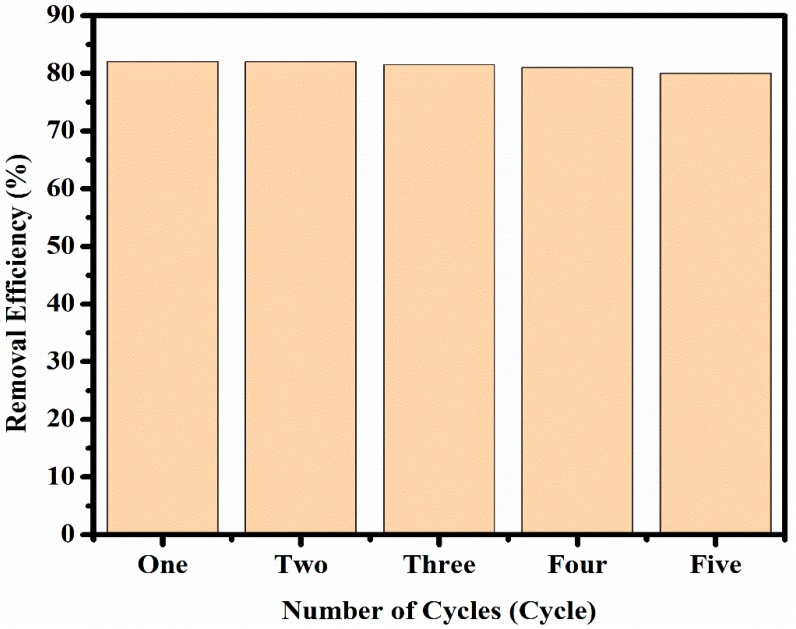
The removal efficiency of Pd/CNF as catalysts for MO degradation remained stable (about 83%) throughout five consecutive cycles of filtration and reuse (after 3 h).

In general, Pd NPs size and Pd quantity were in the same range or even higher than what we reported in this study. NaBH_4_ concentration as well as the catalyst/dye ratios was also higher in all the mentioned studies (in comparison to our system). The degradation ratio was found to be in the range of 90% as detailed in Table 3. The comparison between the performance of our catalyst and the literature demonstrated that the obtained Pd/C materials are so promising and further works are in progress in order to apply our Pd/C catalysts for the degradation of other dyes in the same conditions. We should note, in addition, that the supported nature of our Pd NPs allowed an easy regeneration, as is demonstrated in Figure 8.

Decolorization [1,3,4,77] is the main visible indicator of dye degradation mechanism; normally colored alteration reflect this process, because dyes do not only adsorb light in the visible range (400–750 nm), but also contain at the minimum one chromophore (such as Nitro and Azo groups), and one auxochromes −COOH or −OH (for color enhancement). If any of these constituents is lost, the color of the dye will be also lost. MO in its molecular structure is formed by an azo group in conjugation with benzene rings and a sulphonic acid moiety, this conjugation imparts an orange colour to the dye. Typically, in aqueous solution, sodium borohydride releases hydrogen in presence of a catalyst. The liberated H_2_ reduces the azo group of methyl orange first to imine and finally to the amine stage [76], where the H_2_ gas is chemisorbed on the NP surface, which eventually reduces MO and breaks the conjugation through the diazo (N=N) bond. Consequently, MO loses its color gradually [77].

Indeed, the pH of the solution is one of the significant parameters for the catalytic dye degradation, for example, as it can influence dye reaction and dye adsorption onto the semiconductor surface in the photocatalysis case, where the catalyst surface charge depends on the pH of a given solution. [3,83]

Moreover, the azo linkage (–N=N–) is mainly vulnerable to electrophilic attack by hydroxyl radical(-OH). Riga et al. reported that dye decolorization and degradation rate increases with increasing pH [84].

Furthermore, sodium borohydride involves the electron donor. As mentioned by Li et al. [14], the catalytic efficiency tended to be enhanced based on an optimized concentration of NaBH_4_, as the high-nucleophilicity BH_4_^-^ anions could be absorbed, and where a high concentration of local electrons on the active-sites, namely Pd NPs surface could strongly lead to an increasing of the degradation efficiency. The rate of degradation increases as the pH of the solution decreases, which can be explained by faster hydrolysis of BH_4_- to active neutral borane species at lower pH and thus accelerating the catalytic activity. [85] This requirement of spontaneous hydrolysis of borohydride before its catalytic activation by metal nanoparticles was extensively investigated by Choi et al. [86].

In other word, the electron-rich conjugated aromatic system, -NH and –N= bonds will be helpful to attract the aromatic dyes, based on Lewis acid-base interaction, H-bonding or π-electronic interaction, thus, facilitate dye reduction/degradation [85].

Hence, the Pd/C removal efficiency was analyzed based on the decolorization ratios of MO in aqueous solution at different intervening time. A UV–Vis spectrum, shown in Figure 7, describes the gradually reduction of MO, where the maximum peak at 463 nm was decreasing steadily and finally reached almost its minimum value after 240 min (after 4 h) indicating its removal: 98.9% of MO were reduced by Pd/C catalyst.

In addition to this colorful-physical phenomena recognizable by naked eye, chemically the fate of MO is well-known based on different studies: MO is firstly stuck on the catalyst surfaces [65] and then react with active radicals to finally degrade through many intermediate chemical products like dimethylamine group, hydroxyl group connected with the chromophore group and further oxidization takes place to produce CO_2_, H_2_O and other inorganic ions until total mineralization [73,74].

The recovering ability also seems promising. To assess whether Pd/CNFs could be recycled for dye reduction with NaBH_4_, the catalysts used for the test with MO were recovered simply by filtering with filter paper, washed with milli-Q water, dried at 60 °C, and used again for five times without any obvious activity loss (Figure 8). The activity was investigated after 180 min of dye–catalyst contact.

These results indicated that Pd/CNFs are a sustainable catalyst for dye degradation. The complete process is described in Figure S1 which summarize the whole experiment.

## 4. Conclusions

In this work, advanced Pd/CNFs nanocomposites were successfully synthesized for catalytic applications by combining electrospinning and ALD. Several characterizations were performed, such as SEM, TEM, XRD and EDX, and confirmed the deposition of Pd NPs on CNFs, which was obtained after carbonization of the electrospun PAN NFs. These high-surface Pd/CNFs displayed an outstanding performance for green-removal organic pollutant. This activity was exemplified by the catalytic degradation of an organic dye (MO) in the presence of sodium borohydride, where up to 98.9% of MO was removed. Based on the theoretical adsorption study, the best fittings were described by the Freundlich and pseudo-first-order models. This suggests that first, adsorption took place at the catalyst surface, before MO degradation due to the effect of sodium borohydride. Furthermore, MO adsorption and degradation using Pd/CNFs as effective catalysts is likely to occur as a multilayer adsorption process. In general, the prepared catalysts worked well to catalyze MO reduction in the presence of NaBH_4_ as reducing agent. At the end of the first MO adsorption and degradation cycle, the catalyst could be easily recovered from the reaction solution and was reused several times without any remarkable change in its activity. This study brings some insights into the potential of Pd/CNFs as promising efficient and long-lasting catalysts for toxic organic dye reduction in wastewater and environment recovery.

## Figures and Tables

**Table 1:** The adsorption isotherm parameters for MO adsorption according to the linear Langmuir and Freundlich models.

Model	Parameter		Value
Langmuir	Pd/C	q_max_ (mg/g)	104
		K_L_ (L/mg)	0.0960
		R^2^	0.932
	C	q_max_ (mg/g)	83
		K_L_ (L/mg)	0.0326
		R^2^	0.922
Freundlich	Pd/C	K_F_ ((mg/g)(L/mg)^1/n^)	10.60
		n	1.41
		R^2^	0.998
	C	K_F_ ((mg/g)(L/mg)^1/n^)	3.10
		n	1.17
		R^2^	0.950

**Table 2:** Kinetics parameters for MO adsorption onto Pd/CNF and CNF samples calculated with the pseudo-first-order and pseudo-second-order models.

Model	Parameter		Value
Pseudo-first-order	Pd/C	q_e_ (cal) (mg/g)	30.7
		K_1_ (1/min) × 10^−2^	1.7
		R^2^	0.92
	C	q_e_ (cal) (mg/g)	17.0
		K_1_ (1/min) × 10^−3^	5.1
		R^2^	0.98
Pseudo-second-order	Pd/C	q_e_ (cal) (mg/g)	144.9
		K_2_ (g/(mg·min)) × 10^−6^	5.0
		R^2^	0.67
	C	q_e_ (cal) (mg/g)	28.1
		K_2_ (g/(mg·min)) × 10^−6^	124.9
		R^2^	0.86

**Table 3:** Literature review on the degradation of dyes using Pd NPs as catalysts.

Catalyst	Catalyst Concentration (M)	Dye	Pd Size (nm)	(wt% Pd)	Degradation Time (Optimal)	Removal Efficiency (%)	Ref.
Pd supported on Carbon nanotubes	1.879 × 10^−3^	Methyl orange	15 to 25	17.7	60 min	99.31	[1]
Pd/Fe_3_O_4_-PEI-RGO	0.5 × 10^−7^	Methylene Blue	4.5	1.9	10 min	99	[14]
Pd/porous polyurea microsphere	0.52 × 10^−4^	4-NP nitrophenol	3.5	1.76	6 min	96	[79]
Pd/Carbon nanospheres	0.018	4-Nitrophenol	7	1.3	49 min	95	[80]
Pd NPs	9.42 × 10^−3^	Congo Red CR	12	9.2	14 min	95	[81]
Pd NPs	9.42 × 10^−3^	Sunset Yellow	21	9.2	9 min	97	[81]
Pd NPs	9.42 × 10^−3^	Methyl Orange	12	9.2	12 min	96	[81]
Pd NPs	9.42 × 10^−3^	Tartrazine	12	9.2	12 min	96	[81]
Pd/Carbon Cloth	1.688 × 10^−3^	4-Nitrophenol	95	1.9	7 min	94	[82]
Pd/Carbon Cloth	1.688 × 10^−3^	Congo Red	95	1.9	2 min	94	[82]
Pd/Carbon Cloth	1.18 × 10^−3^	Methylene Blue	95	1.9	3s	94	[82]
Pd/CNFs	0.016	Methyl Orange	7	8.3 ± 3.2	240 min	98.9	This work

## Supplementary Materials

The following are available online at https://www.mdpi.com/1996-1944/13/8/1947/s1, Figure S1: Summary of the whole original mechanism.
