# Supplementary material for: Palladium/Carbon Nanofibers by Combining Atomic Layer Deposition and Electrospinning for Organic Pollutant Degradation
Source: Materials (Basel). 2020 Apr 21;13(8):1947. doi: 10.3390/ma13081947 (PMC7215890; doi:10.3390/ma13081947)
Supplement: Supplementary file 1 [file materials-13-01947-s001.pdf]

materials

Supplementary Materials: Palladium/Carbon Nanofibers by Combining Atomic Layer Deposition and Electrospinning for Organic Pollutant Degradation

Melissa Najem, Amr A. Nada, Matthieu Weber, Syreina Sayegh, Antonio Razzouk, Chrystelle Salameh, Cynthia Eid and Mikhael Bechelany *

Scherrer Equation

The Scherrer equation (Equation S1) can be written as:

$$\tau = \frac{K\,\lambda}{\beta\ cos\theta} \quad \text{(S1)}$$

where:
τ is the nano-mean crystallite size;
K is a dimensionless shape factor, with a value close to unity (typical value of about 0.9)
λ is the X-ray wavelength;
β is the line broadening at half the maximum intensity (FWHM) in radian;
θ is the Bragg angle.

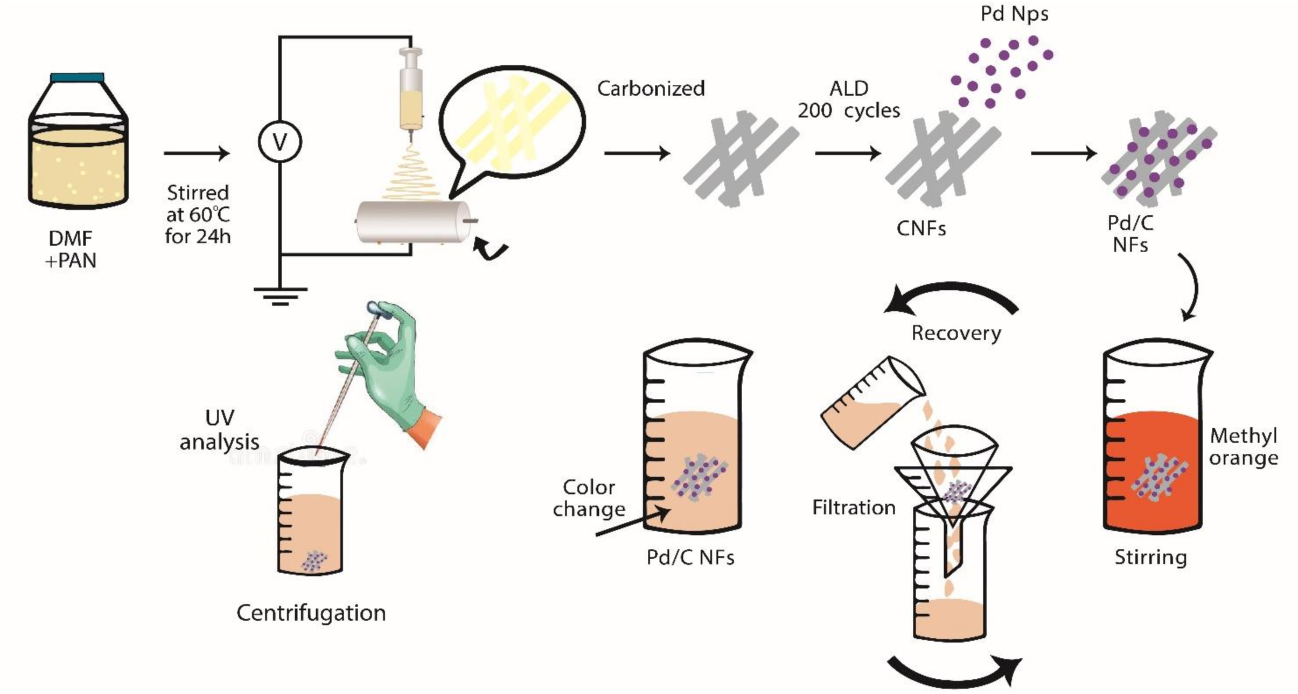

Figure S1. Summary of the whole original mechanism.

© 2020 by the authors. Licensee MDPI, Basel, Switzerland. This article is an open access article distributed under the terms and conditions of the Creative Commons Attribution (CC BY) license (http://creativecommons.org/licenses/by/4.0/).
